# Characterizing infectious disease progression through discrete states using hidden Markov models

**DOI:** 10.1371/journal.pone.0242683

**Published:** 2020-11-20

**Authors:** Kristina M. Ceres, Ynte H. Schukken, Yrjö T. Gröhn

**Affiliations:** 1 Department of Population Medicine and Diagnostic Sciences, College of Veterinary Medicine, Cornell University, Ithaca, NY, United States of America; 2 Department of Animal Sciences, Wageningen University, Wageningen, The Netherlands; Universitatsmedizin Greifswald, GERMANY

## Abstract

Infectious disease management relies on accurate characterization of disease progression so that transmission can be prevented. Slowly progressing infectious diseases can be difficult to characterize because of a latency period between the time an individual is infected and when they show clinical signs of disease. The introduction of *Mycobacterium avium ssp*. *paratuberculosis* (MAP), the cause of Johne’s disease, onto a dairy farm could be undetected by farmers for years before any animal shows clinical signs of disease. In this time period infected animals may shed thousands of colony forming units. Parameterizing trajectories through disease states from infection to clinical disease can help farmers to develop control programs based on targeting individual disease state, potentially reducing both transmission and production losses due to disease. We suspect that there are two distinct progression pathways; one where animals progress to a high-shedding disease state, and another where animals maintain a low-level of shedding without clinical disease. We fit continuous-time hidden Markov models to multi-year longitudinal fecal sampling data from three US dairy farms, and estimated model parameters using a modified Baum-Welch expectation maximization algorithm. Using posterior decoding, we observed two distinct shedding patterns: cows that had observations associated with a high-shedding disease state, and cows that did not. This model framework can be employed prospectively to determine which cows are likely to progress to clinical disease and may be applied to characterize disease progression of other slowly progressing infectious diseases.

## 1. Introduction

Slowly progressing infectious diseases, like tuberculosis in humans and animals, HIV/AIDS in humans, and Johne’s disease in cattle, are difficult to characterize because they are often associated with a latency period between the time an individual is infected and when they show clinical signs or symptoms of disease. Treatment efficacy and transmission dynamics are dependent on the state of infection, and understanding an individual’s infection state, and probability of transitioning among infectious states at the population level, can help provide better treatment protocols and targeted epidemiologic interventions. For example, only patients with active tuberculosis infections are likely to transmit *Mycobacterium tuberculosis* to others; however, targeting latently infected individuals that have a high probability to progress to active disease with preventative therapeutics could prevent transmission from occurring [1].

Johne's disease is caused by *Mycobacterium avium subsp*. *paratuberculosis* (MAP) and is characterized by a chronic granulomatous enteritis leading to diarrhea, wasting and eventually death. Johne’s disease is considered an important production disease, especially in the dairy industry, and is estimated to cost 200 million dollars per year in production losses and early culling in the United States [2]. MAP has also been implicated as a possible cause or contributing factor to Crohn’s disease in humans; therefore, zoonotic MAP transmission may be relevant to public health [3]. Johne's Disease is pervasive in dairy farms in the United States. In 2007, 68 percent of dairy operations tested positive for MAP in at least one environmental sample [4]. MAP is mainly transmitted among cattle through the fecal-oral route and animals can become infected by eating contaminated material from their environment [5]. Calves are more susceptible than other age groups, and often become infected either in-utero or soon after birth; however, they may not show clinical signs of disease for 2–5 years [5–7]. MAP can also survive for up to a year in certain environmental conditions, which could allow new infections even after an infected animal is removed from the herd [5,8]. Due to, among other reasons, persistence in the environment and a long latency period before infected animals show clinical signs, Johne's disease is very difficult to control.

In order to effectively control Johne's disease, it is essential to understand how the infection progresses. It is currently hypothesized that animals in certain disease states transiently shed MAP in their feces, and it is known that some animals shed much more MAP than others [9]. Schukken et al. observed two distinct patterns of disease progression among infected animals [10]; animals that progressed to a high shedding disease state, and those that controlled infection. Furthermore, Mitchell et al. observed that most animals that shed MAP intermittently never progress to a high shedding state, but those that do progress maintain a consistent high level of shedding [11].

Johne's disease infection dynamics have been studied extensively using compartmental and agent-based models [12–14]; however, these models contain a large number of parameters, and it is sometimes necessary to rely on assumptions instead of data in order to fully parameterize the model. In order to study Johne's and other disease dynamics it is usually necessary for the investigator to divide disease progression into a series of discrete states and determine reasonable rates of movement between the states to parameterize the model. Some model parameters can be estimated using field data, but parameters regarding transition among disease states are less obviously estimable, especially in the case of Johne's disease, where animals can maintain an infectious state for years without any visible signs of disease. With slowly progressing diseases like Johne's, it may be difficult or in many animals impossible to observe direct signs of subclinical disease states, so determining parameters for rates of transition among disease states is non-trivial. Hidden Markov models (HMMs) offer one solution to the problem of estimating these parameters by using unsupervised machine learning to estimate model parameters including disease state transition rates. A validated HMM can also be used as a diagnostic to predict disease progression outcomes in infected cows.

We constructed sequences of MAP fecal culture results from repeated samples of individual animals sampled biannually up to 6 years from three Northeast US dairy herds, and used these sequences to infer the underlying hidden Johne's disease states in a HMM framework. Our objectives were to estimate disease state and transition parameters among infected cows, and to determine if two or more types of disease progression existed. We hypothesized that the most probable path through hidden states identified by posterior decoding would show two distinct most probable state progression paths that represent separate MAP infection progression types.

## 2. Methods

### 2.1 Data description

**Fig 1:**
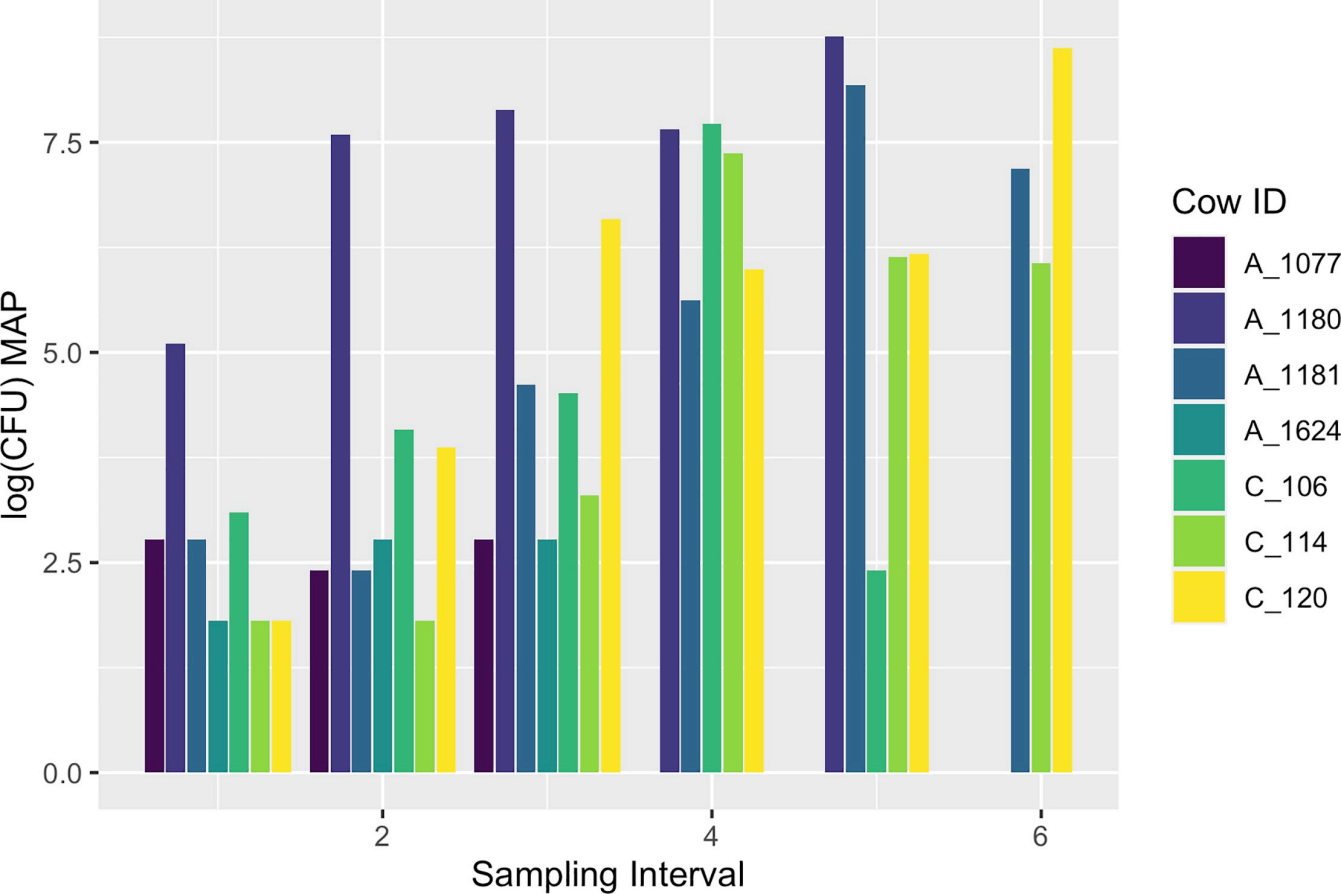
MAP fecal shedding patterns from five seven cows. Shedding patterns were variable among individuals. The number of samples taken from each cow was also variable, ranging from 2 to 11 samples. The variation in both CFU count and number of samples may be related to disease progression, as animals with high CFU counts may be culled early due to clinical MAP infection related production losses.

Fecal samples were taken from all adult animals on three northeastern farms between 2004 and 2009 biannually, totaling 6530 samples from 1714 cows as part of the Regional Dairy Quality Alliance Management (RDQMA) program [15]. The overall period prevalence was 5.9%, and fecal shedding was variable among infected cows ranging from 0 to 6360 colony forming units (CFU). Cows were sampled between 2 and 11 times with only one cow sampled 11 times over 5.5 years. Fecal samples from each cow were placed in four 15 ml solid medium slant tubes, and were processed and cultured as described previously [15,16]. The sum of CFU cultured from the four tubes was multiplied by 5.3 to account for dilutions, and the log base 10 of the resulting quantity was used as the observed CFU count for model fitting. Positive samples were defined as greater than 0 CFU in a fecal sample. Samples were taken on average 15 weeks apart with a median of 14 weeks apart; however, sampling intervals ranged from 1 to 40 weeks. To avoid misclassification of healthy cows as infected, we only included cows that had at least two positive fecal culture samples out of three consecutive samples. Samples that were not readable due to contamination with other bacterial growth or fungal growth were removed from the analysis. After removing samples that did not meet inclusion criteria, we were left with 129 observations. Fecal shedding patterns of seven representative cows are shown in Fig 1.

### 2.2 Model description

A HMM is a probabilistic model which describes the transition through unobserved states in a Markovian system. Although the hidden state of the system cannot be observed directly, information about the state can be inferred indirectly through model emissions. This framework is useful for modeling MAP infection progression because the underlying disease state is not readily observable, but MAP shedding in feces can be easily measured and can be used in the HMM framework as model emissions. We developed four candidate continuous time HMMs to model MAP infection progression in dairy cows. Since samples were taken at irregular intervals, we chose to model disease progression using a continuous time HMM framework as opposed to a discrete time framework. In a continuous time HMM, both the hidden state at the time a sample was taken and the number and types of transitions between hidden states between two consecutive observations are unknown [17]. Continuous time HMMs are defined by a vector of initial hidden state probabilities, ***π***_**0**_ that define the probability of being in each state at time 0, a state transition rate matrix **Q** that defines the rate of transition among hidden states, and emission probability distributions, ***E***. The emission probability distributions, which are defined as the probability of observing a log_10_ CFU given an underlying hidden disease state k, were modeled using gamma distributions, *E*_*k*_~*Gamma*(*α*_*k*_,*θ*_*k*_), where *α*_*k*_ and *θ*_*k*_ are gamma distribution shape and scale parameters, respectively. The probability of transitioning from state j to state k depends on the interval of time t and is defined as *P*_*jk*_(*t*) = *e*^*Qt*^.

**Fig 2:**
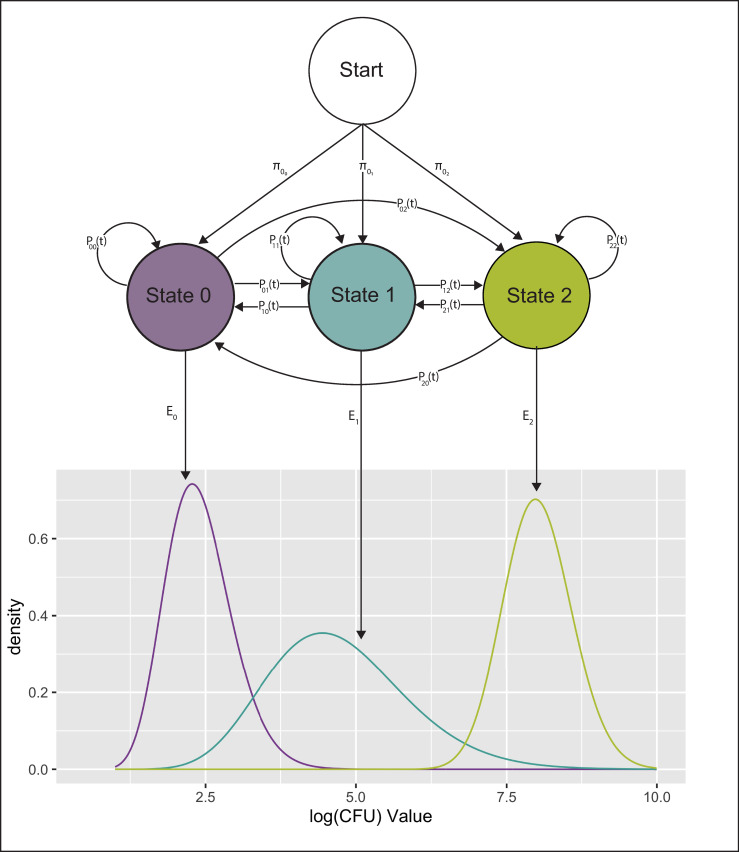
Diagram of hidden Markov model 1. This diagram represents a hidden Markov model with three hidden states labeled 0, 1, and 2. The model is shown with initial state probabilities, *π*_0_, time dependent transition probabilities, *P* and emissions, *E*, which have gamma distributions.

The number of true disease states is unknown, but models with very large state spaces may not provide a useful representation of MAP infection from veterinary standpoint since as the number of states increases the differences in disease phenotype between states may diminish, and states without a remarkable associated change in disease phenotype do not have obvious clinical relevance. Additionally, the potential model state space that can be explored is limited by our relatively low sample size of cows with MAP infection. Therefore, we chose to create a candidate model set including models with two to five hidden states to capture clinically relevant HMM structures. A graphical description of an example three hidden state model is shown in Fig 2. In Fig 2, the state labels “0”, “1” and “2” represent disease states associated with low, moderate, and high CFU counts, respectively.

### 2.3 Parameter learning

Maximum likelihood estimates for model parameters, *π*_0_, *Q*, *α*, *θ*, were computed using a modified Baum-Welch expectation maximization (EM) algorithm developed for continuous time HMMs by Liu et al. [17]. We used the “Expm” method, which relies on the matrix exponential of an auxiliary matrix *A* to calculate EM estimates of model parameters. The auxiliary matrix, $A=\left(\begin{array}{cc} Q & B\\ 0 & Q \end{array}\right),$ was calculated for each state *i*, and edge *i*,*j*. B is a square matrix with the same dimensions of Q and has 1 in the (*i*,*j)* position and 0 elsewhere. This method was determined to be the most robust EM algorithm of the three presented by Liu et al. At each iteration of Expm EM, gamma shape and scale parameters were updated using the Newton-Raphson method, which is further described in the S1 File. The algorithm guarantees an increase in model likelihood with each iteration, and was terminated when the change in likelihood was less than 0.001. All algorithms were implemented in Python 3.7.0 using open source libraries: Scipy [18], NumPy [19], and pandas [20].

### 2.4 Initial parameterization and model selection

Each model was initialized with 250 sets of parameter values drawn from uniform distributions shown in Table 1. Initial state probabilities ***π***_**0**_ were normalized such that each set of initial state probabilities summed to 1. The fit of each model state structure was assessed using Akaike’s information criterion (AIC) calculated as 2*p*−2*log*(*L*), where *L* is the model likelihood and *p* is the number of parameters estimated in a specific model.

**Table 1:** Descriptions and initial values of parameters in three hidden state models.

*Parameter*	*Description*	*Initial Value*
$\pi_{0_{k}}$	Probability of starting in each hidden state k	Unif(0,1)
*α*_*k*_	Gamma distribution shape parameter for state k	Unif(1,20)
*θ*_*k*_	Gamma distribution scale parameter for state k	Unif(0.01, 1)
*q*_*jk*_	Transition rate from state j to state k	Unif(0.01,5)

Four hidden Markov model structures with two, three, four or five hidden states were initialized with 250 sets of random parameter values drawn from uniform distributions. We chose parameter values for transition rates and emission distributions that would produce reasonable state transition probabilities and gamma distributions to fit the observed CFU values. We allowed the initial state probabilities to range from 0 to 1 reflecting our limited knowledge on the proportion of animals in each underlying disease state.

### 2.5 Bootstrap confidence intervals

The CFU shedding dataset of 129 cows was sampled with replacement to generate 1000 bootstrap samples of the same size as the original dataset using the resample function from sklearn [21]. Model transition and emission parameters were estimated for each sample using the initialization parameter combination that produced the lowest AIC score. 95% confidence intervals were calculated for each transition and emission probability using the 2.5 and 97.5 percentiles of the parameter values generated using the bootstrap samples.

## 3. Results and discussion

### 3.1 Model likelihood and AIC

The AIC values for each model type (two, three, four, five states) is shown in Table 2.

**Table 2:** Model comparison using AIC.

*Model*	*p*_*i*_	*log(L*_*i*_*)*	*AIC*_*i*_	*Rel*. *L*_*i*_	*w(AIC)*_*i*_
3 State	14	-95.83	**219.66**	1	0.993
2 State	7	-107.86	229.72	6.5 E -3	0.006
4 State	23	-106.24	258.48	3.7 E -9	3.7 E -9
5 State	34	-104.38	276.76	4.0 E -13	4.0 E -13

*Rel*. *L*_*i*_ represents the relative log likelihood for model *i* and is calculated as $exp(\frac{AIC_{min}-AIC_{i}}{2})$ where is *AIC*_*min*_ the minimum AIC value in the candidate set. *w(AIC)*_*i*_ is the AIC weight for model *i* and is calculated as $\frac{Rel.L_{i}}{\sum_{k}Rel\;L_{k}}$. *p*_*i*_ is the number of parameters in model *i*, and *log(L*_*i*_*)* is the log likelihood for model *i*.

The three state model had the lowest AIC and had the largest AIC weight so it was determined to be the best fitting model out of the four types tested to minimize information loss. This suggests that the three state model fits the data best with our limited sample size, but it is possible that with a larger dataset a different state structure would be preferred. For example, if there were more consecutive samples taken for each cow over a longer period of time, there may be stronger support for a model with a larger number of states as this dataset may contain finer scale changes in shedding patterns that were not evident in our comparatively sparse dataset. On the other hand, factors such as individual heterogeneity and outliers may lead AIC to favor models with larger state spaces when smaller state spaces are more biologically realistic [22]. Although the three state model had the lowest AIC, since AIC may favor larger state spaces, and since both the two and three state models had AIC weights greater than zero, for the remainder of this paper will focus on results for both the two and three state models. The initial parameter combination that produced the lowest AIC value for the two, three, four and five state models are shown in S1 Table. Parameter estimates for the four and five state models are shown in S2 Table.

### 3.2 Parameter estimates

EM estimates for two and three hidden state model parameters are shown in Table 3.

**Table 3:** Transition and emission probability estimates.

*Parameter*	*Description*	*Estimate*	*95% CI*
Two state models			
*π*_0_	Probability of starting in state 0	0.45	(0.30, 0.64)
*α*_0_	Gamma distribution shape parameter for state 0	17.56	(4.81, 26.33)
*α*_1_	Gamma distribution shape parameter for state 1	17.86	(13.86, 184.37)
*θ*_0_	Gamma distribution scale parameter for state 0	0.14	(0.09, 0.82)
*θ*_1_	Gamma distribution scale parameter for state 1	0.39	(0.04, 0.48)
*α*_0_*θ*_0_	Gamma distribution mean for state 0	2.44	(2.30, 3.95)
*α*_1_*θ*_1_	Gamma distribution mean for state 1	6.87	(6.49, 8.03)
*q*_01_	Transition rate from state 0 to 1	0.03	(0.01, 2.90)
*q*_10_	Transition rate from state 1 to 0	2.00e-3	(1.22e-07, 1.02)
Three state models			
*π*_0_	Probability of starting in state 0	0.43	(0.29, 0.62)
*π*_1_	Probability of starting in state 1	0.21	(4.11e-36, 0.39)
*α*_0_	Gamma distribution shape parameter for state 0	19.41	(4.79, 30.07)
*α*_1_	Gamma distribution shape parameter for state 1	16.24	(12.29, 15307.72)
*α*_2_	Gamma distribution shape parameter for state 2	173.20	(36.00, 239.35)
*θ*_0_	Gamma distribution scale parameter for state 0	0.12	(0.08, 0.78)
*θ*_1_	Gamma distribution scale parameter for state 1	0.35	(3.27e-4,0.39)
*θ*_2_	Gamma distribution scale parameter for state 2	0.05	(0.03, 0.20)
*α*_0_*θ*_0_	Gamma distribution mean for state 0	2.40	(2.17, 3.75)
*α*_1_*θ*_1_	Gamma distribution mean for state 1	5.64	(3.83, 6.18)
*α*_2_*θ*_2_	Gamma distribution mean for state 2	7.80	(7.14, 8.12)
*q*_01_	Transition rate from state 0 to 1	0.03	(0.01, 0.98)
*q*_02_	Transition rate from state 0 to 2	1.13e-40	(1.89e-46, 0.48)
*q*_10_	Transition rate from state 1 to 0	4.65e-39	(1.86e-131, 1.46)
*q*_12_	Transition rate from state 1 to 2	0.03	(0.02, 6.15)
*q*_20_	Transition rate from state 2 to 0	3.00e-3	(5.12e-143, 0.60)
*q*_21_	Transition rate from state 2 to 1	1.50e-06	(1.50e-8, 1.49)

Parameter estimates for the two and three hidden disease models were generated using the modified Baum-Welch Expectation Maximization algorithm and bootstrap confidence intervals, using initial values in S1 Table.

**Fig 3:**
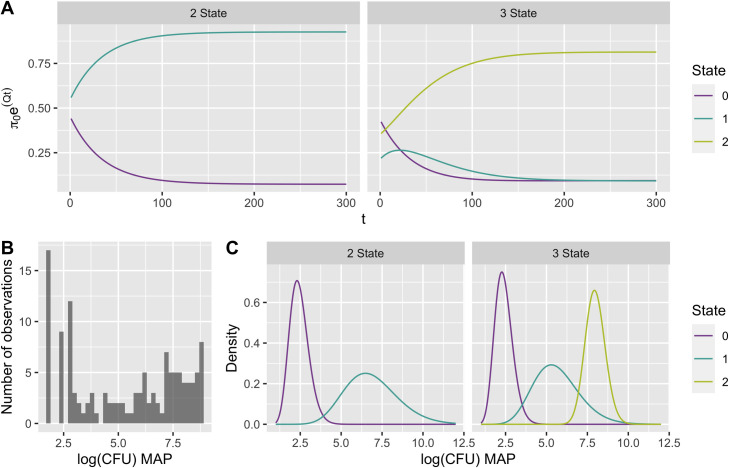
Transient distributions and emission distributions in two and three hidden state models. The transient distribution for each Markov chain was calculated for time t = 0,..,300 weeks (A). Fitted emission distributions are shown in (C), and the observed data are shown in grey (B). The number of random samples used to create each plotted state emission distribution was proportional to the stationary distribution value for that hidden state.

Transient distributions of the Markov chain are shown in Fig 3A. A histogram of the transformed CFU data is shown in Fig 3B and fitted emission distributions for the two and three state models are shown in Fig 3C. The CFU count data shows a large number of low CFU observations, fewer mid-range CFU counts between 3 and 5 CFU and a larger number of observations between 5 and 9 CFU. The CFU count data are well approximated by the fitted gamma emissions, which show higher densities for low and high CFU counts than for mid-range CFU counts.

The stationary distributions *π*_*s*_ are calculated by solving *π*_*s*_*Q* = 0, and can be approximated by the equilibrium distribution shown in Fig 3A, where for large *t*, $\pi_{0}^{-Qt}\approx \pi_{s}$. The stationary distribution for the two state model was *π*_*s*_ = [0.07,0.93], and for the three state model was *π*_*s*_ = [0.09,0.09,0.81]. For both the stationary distributions in the two and three state models, there was a very high probability of being in the highest disease state (state 1 in the two state model and state 2 in the three state model). Thus, the majority of cows are expected to reach a disease state associated with high CFU shedding; however, a minority of cows are expected to have observations associated with a low-shedding disease state.

**Fig 4:**
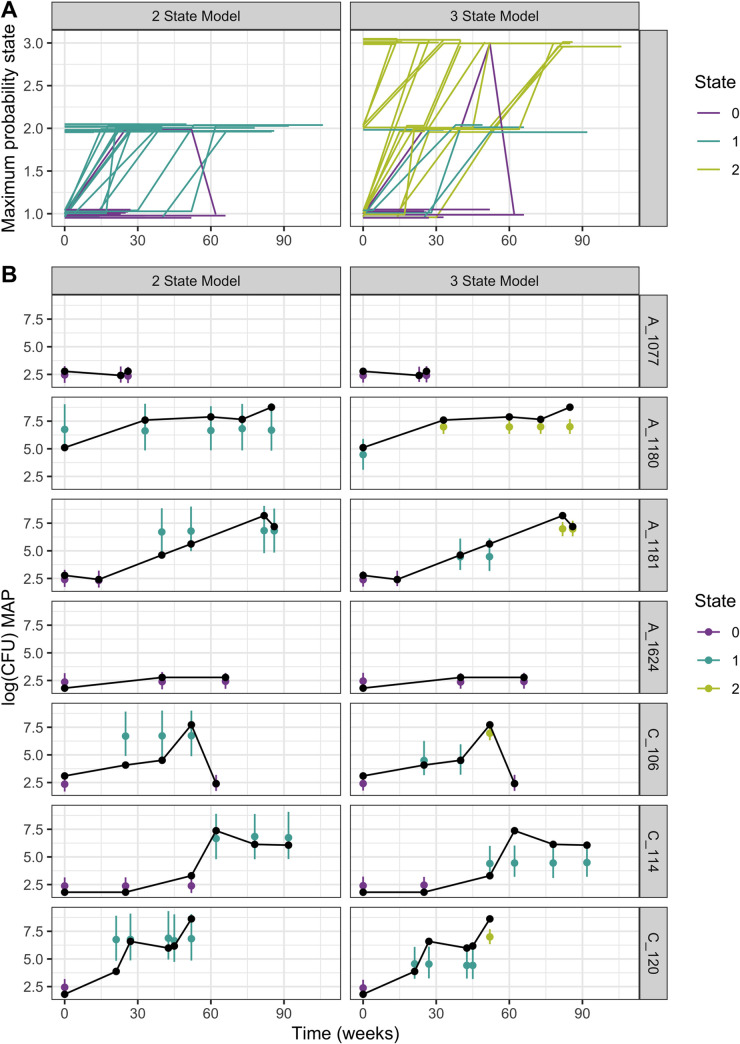
Progression patterns through disease states. Progression patterns of individual cows through disease states estimated using posterior decoding are shown for both the two state and the three state models (A). The color of the line indicates the state with the highest posterior probability at the last observation. The lines were jittered vertically so that overlapping lines could be distinguished. The empirical and predicted log(CFU) MAP value from seven representative cows shows variability in fecal shedding over the sampling period, with some cows shedding very high CFU counts over the sampling period and others maintaining a lower CFU count (B) The median, and 10–90 percentile range for the predicted log(CFU) distributions are shown as points and bars, respectively. The predicted CFU values are colored according to the fitted state emission distribution for each sample. The empirical log(MAP) CFU counts are shown in black.

The sojourn time before transitioning between hidden states is exponentially distributed with parameter -*q*_*ii*_, where *q*_*ii*_ is a diagonal element of the Q matrix. In both the two and three state models, the probability of remaining within a state is much higher than transitioning between states. The average sojourn time for state 0 in the two state model was 37.7 weeks, and the average sojourn time in the three state model was 38.3 weeks for state 0 and 37.8 weeks for state 2; therefore, on average cows remained in a low CFU-emitting disease hidden state for about 38 weeks before transitioning to a higher CFU-emitting disease state. Unlike the two state model, the three state model contains an intermediate state (state 1) that is associated with a moderate level of MAP shedding. In the three state model, the transition rate from state 0 to 1 is much higher than the transition rate from state 2 to 1, and the transition rate from state 1 to 2 is much higher than the transition rate from state 1 to 0. Similarly, in both the two and three state models, the transition rate between lowest shedding disease states to the highest shedding disease state was much higher that the transition rate from a high shedding disease state to lower shedding disease states. Together, this indicates that transition from any higher shedding state to a lower shedding state is less likely than either remaining in the current state or transitioning to a higher shedding disease state, which is consistent with the known progression of MAP infection from subclinical infection associated with low MAP shedding to clinical Johne’s disease associated with high MAP shedding. In addition to transitioning from low shedding to high shedding states, transition rate estimates in the three state model suggest that it is possible to transition from state 2 to state 0, and that this transition is more likely than transitioning from state 2 to 1. This type of transition may exist in an intermittent high shedding progression pattern, where cows have observations associated with a high shedding disease state flanked by observations associated with a low shedding disease state; however, this pattern is only clearly demonstrated by one cow, C_106, shown in Fig 4B, where between 50 and 65 weeks there is a transition from state 2 to state 0.

The maximum posterior probabilities of a cow being in a particular disease state at each sampling time were constructed to visualize progression patterns of individual cows, and these individual progression patterns are shown in Fig 4A. The observed and predicted log(CFU) MAP values for representative cows is shown in Fig 4B. The predicted log(CFU) MAP values were generated by taking 1000 random samples from the fitted gamma emission distributions from the state with the highest posterior probability for each observation. The majority of the predicted log(MAP) 10–90 percentiles overlapped the observed log(MAP) values; however, the three state model 10–90 percentile range predictions were narrower and overlapped observed values more often than the two state model predictions indicating that the three state model fit the data better, especially for intermediate log(CFU) values.

In both the two and three state model structures, the majority of cows had increasing fecal CFU counts over the sampling period; however, a small group of cows maintained a low CFU count. This group, labeled as having an observation at the last sampling interval in the lowest disease state in Fig 4A, represents the proportion of cows with consecutive low CFU counts that did not progress to a high-shedding disease state. The stationary distributions of the Markov chains and the differential posterior decoded patterns demonstrate that there are at least two types of MAP infection progression patterns, where most cows progress to a disease state associated with higher MAP shedding in both the two and three state models. These “progressors” shed higher MAP loads over the course of their infection than “non-progressors” that remain in lower CFU-emitting disease states. This suggests that there may be a minority of infected cattle that do not shed large amounts of MAP, and thus may play a smaller role in the transmission chain within a farm. If these animals are high producers, it may be reasonable to keep them on the farm despite their positive MAP infection status.

### 3.3 Potential factors related to disease progression path

Our study provides additional evidence to support that at least two progression patterns exist among cows infected with MAP. Previous studies have suggested that age at time of infection plays a role in disease progression type [11]; however, we were not able to determine the age at the time of infection since all animals were infected naturally, and we only had samples from adult animals. There is also evidence that host immune factors are related to Johne’s disease progression in cattle and other species. De Silva et al. found that high fecal MAP DNA quantity and lower interferon gamma response early in life were positively associated with progressing to clinical disease in sheep, suggesting that early immune response may play a role in disease progression path [23]. Koets et al. found that cows with mutations in the Toll-like receptor 2 gene may show an increased macrophage activity, increased T cell activation and reduced susceptibility to MAP infection [24]. Future research incorporating disease path progression using HMM and immune monitoring could lead to breeding cattle that are resistant to progression to the state of high shedding.

### 3.4 Future directions and conclusions

Mathematical modeling has been an important tool to study Johne’s disease control due to the complex nature of the slowly progressing disease dynamics and farm dynamics. Some previous modeling studies have included non-progressing and progressing patterns through discrete disease states [13,25]; however no study has estimated probabilities of transition among those disease states, or the probability of shedding CFU count given membership in a specific disease state. The addition of state transition probabilities from our model can improve the accuracy of predicted progression patterns in mathematical models, and the addition of emission probability estimates to simulation models can more accurately model disease transmission dynamics. In addition to the utility of our model estimates in simulation studies, our HMM can also be used for disease progression path prediction on infected farms. After validation in an external population, the model can be incorporated into Johne’s disease control programs to predict which progression paths are more likely given a series of fecal culture samples from individual cows. With this knowledge, and with knowledge from future simulation studies that take advantage of a multi-progression path disease structure, farmers can make more informed decisions on which animals to cull depending on their predicted disease paths.

The proposed model includes simplifying assumptions that do not fully characterize the complex nature of disease progression. One such simplification is creating discrete disease states and fecal shedding states, where in reality disease progression is a continuous process through many possible disease states. Our ability to resolve fine-scale differences between disease states is limited by the intervals between samples, and by the number of samples taken from each cow. It is possible that including covariates such as milk production data or sampling cows more frequently and over a longer period of time could provide a more accurate description of disease progression. Our analysis may also underestimate the true prevalence of MAP infected cows at each sampling interval because some positive animals may not have been identified using fecal culture. Other diagnostic tests exist relying on serological data, but fecal culture may be the most reliable marker for disease progression [26]. Another potential limitation is that expectation maximization may identify local maxima, and may not find global maxima; however, initializing the model with a range of parameter values allowed us to explore a variety of potential maxima. Additionally, our analysis may be influenced by survival bias because cows with more advanced MAP infection may produce less milk than cows with subclinical infection, which results in higher culling rates in lower producing cows. This could result in an underrepresentation of severe cases with higher MAP CFU counts than would be expected if no culling decisions were related to MAP infection stage. Although we expect this survival bias to reduce the expected sojourn time of the hidden states associated with higher CFU counts, we believe our data are representative of MAP infection patterns on typical dairy farms that make culling decisions based on milk production statistics. Lastly, the parameter estimates generated by the model reflect progression patterns among cows included in our study, and the model needs to be validated in an external population to determine if the progression patterns are true of all infected animals.

Johne’s disease is difficult to control due to a number of factors including its slow progression and is extremely difficult to eliminate from a farm due to environmental persistence. We determined that there are at least two distinct progressing patterns, non-progressing and progressing, among infected animals in the three herds studied. Non-progressors are likely to have low fecal MAP CFU counts associated with a low-shedding disease state, whereas progressors are more likely to transition from low to high disease state, which is associated with a high (> 600) CFU count. Parameters from this model can be used to inform disease simulation models to test control strategies that include methods targeted at managing progressors. After model validation, this model can also be used on infected farms to predict future disease states after a relatively small number of observed fecal culture counts. This modeling framework can be used to classify progression in other slowly progressing infectious diseases.

## Supporting information

S1 Table Initial parameter values. These input parameter values produced the lowest AIC values for the two and three state models.
(DOCX)

S2 Table Four and five state model parameter estimates. Parameter estimates for the two and three hidden disease models were generated using the modified Baum-Welch Expectation Maximization algorithm.
(DOCX)

S1 File Description of gamma parameter estimation. This section contains a detailed description of the Newton-Raphson Estimation of gamma emission parameters.
(DOCX)
