# Supplementary material for: Characterizing infectious disease progression through discrete states using hidden Markov models
Source: PLoS One. 2020 Nov 20;15(11):e0242683. doi: 10.1371/journal.pone.0242683 (PMC7678993; doi:10.1371/journal.pone.0242683)
Supplement: S1 Table — These input parameter values produced the lowest AIC values for the two and three state models. (DOCX) [file pone.0242683.s001.docx]

Table S1. Initial parameter values

| *Parameter* | *Description* | *Initial Value* |
| --- | --- | --- |
| Two state models | | |
| $\pi_{0}$ | Estimate for probability of starting in state 0 | 0.44 |
| $\alpha_{0}$ | Gamma distribution shape estimate for state 0 | 4.42 |
| $\alpha_{1}$ | Gamma distribution shape estimate for state 1 | 10.18 |
| $\theta_{0}$ | Gamma distribution scale estimate for state 0 | 0.84 |
| $\theta_{1}$ | Gamma distribution scale estimate for state 1 | 0.45 |
| $q_{01}$ | Transition rate estimate from state 0 to 1 | 4.35 |
| $q_{10}$ | Transition rate estimate from state 1 to 0 | 0.38 |
| Three state models | | |
| $\pi_{0}$ | Estimate for probability of starting in state 0 | 0.28 |
| $\pi_{1}$ | Estimate for probability of starting in state 1 | 0.40 |
| $\alpha_{0}$ | Gamma distribution shape estimate for state 0 | 1.09 |
| $\alpha_{1}$ | Gamma distribution shape estimate for state 1 | 9.88 |
| $\alpha_{2}$ | Gamma distribution shape estimate for state 2 | 19.8 |
| $\theta_{0}$ | Gamma distribution scale estimate for state 0 | 0.42 |
| $\theta_{1}$ | Gamma distribution scale estimate for state 1 | 0.76 |
| $\theta_{2}$ | Gamma distribution scale estimate for state 2 | 0.51 |
| $q_{01}$ | Transition rate estimate from state 0 to 1 | 0.60 |
| $q_{02}$ | Transition rate estimate from state 0 to 2 | 0.20 |
| $q_{10}$ | Transition rate estimate from state 1 to 0 | 2.26 |
| $q_{12}$ | Transition rate estimate from state 1 to 2 | 4.60 |
| $q_{20}$ | Transition rate estimate from state 2 to 0 | 0.99 |
| $q_{21}$ | Transition rate estimate from state 2 to 1 | 0.66 |
| Four state models | | |
| $\pi_{0}$ | Estimate for probability of starting in state 0 | 0.23 |
| $\pi_{1}$ | Estimate for probability of starting in state 1 | 0.29 |
| $\pi_{2}$ | Estimate for probability of starting in state 2 | 0.45 |
| $\alpha_{0}$ | Gamma distribution shape estimate for state 0 | 2.83 |
| $\alpha_{1}$ | Gamma distribution shape estimate for state 1 | 8.65 |
| $\alpha_{2}$ | Gamma distribution shape estimate for state 2 | 13.4 |
| $\alpha_{3}$ | Gamma distribution shape estimate for state 3 | 18.9 |
| $\theta_{0}$ | Gamma distribution scale estimate for state 0 | 0.62 |
| $\theta_{1}$ | Gamma distribution scale estimate for state 1 | 0.97 |
| $\theta_{2}$ | Gamma distribution scale estimate for state 2 | 0.96 |
| $\theta_{3}$ | Gamma distribution scale estimate for state 3 | 0.45 |
| $q_{01}$ | Transition rate estimate from state 0 to 1 | 2.13 |
| $q_{02}$ | Transition rate estimate from state 0 to 2 | 1.15 |
| $q_{03}$ | Transition rate estimate from state 0 to 3 | 2.45 |
| $q_{10}$ | Transition rate estimate from state 1 to 0 | 1.71 |
| $q_{12}$ | Transition rate estimate from state 1 to 2 | 1.11 |
| $q_{13}$ | Transition rate estimate from state 1 to 3 | 1.36 |
| $q_{20}$ | Transition rate estimate from state 2 to 0 | 4.12 |
| $q_{21}$ | Transition rate estimate from state 2 to 1 | 1.02 |
| $q_{23}$ | Transition rate estimate from state 2 to 3 | 3.82 |
| $q_{30}$ | Transition rate estimate from state 3 to 0 | 4.83 |
| $q_{31}$ | Transition rate estimate from state 3 to 1 | 4.01 |
| $q_{32}$ | Transition rate estimate from state 3 to 2 | 1.26 |
| Five state models | | |
| $\pi_{0}$ | Estimate for probability of starting in state 0 | 0.05 |
| $\pi_{1}$ | Estimate for probability of starting in state 1 | 0.14 |
| $\pi_{2}$ | Estimate for probability of starting in state 2 | 0.31 |
| $\pi_{3}$ | Estimate for probability of starting in state 3 | 0.13 |
| $\alpha_{0}$ | Gamma distribution shape estimate for state 0 | 9.69 |
| $\alpha_{1}$ | Gamma distribution shape estimate for state 1 | 11.4 |
| $\alpha_{2}$ | Gamma distribution shape estimate for state 2 | 15.0 |
| $\alpha_{3}$ | Gamma distribution shape estimate for state 3 | 15.0 |
| $\alpha_{4}$ | Gamma distribution shape estimate for state 4 | 18.5 |
| $\theta_{0}$ | Gamma distribution scale estimate for state 0 | 0.74 |
| $\theta_{1}$ | Gamma distribution scale estimate for state 1 | 0.60 |
| $\theta_{2}$ | Gamma distribution scale estimate for state 2 | 0.36 |
| $\theta_{3}$ | Gamma distribution scale estimate for state 3 | 0.22 |
| $\theta_{4}$ | Gamma distribution scale estimate for state 4 | 0.88 |
| $q_{01}$ | Transition rate estimate from state 0 to 1 | 4.79 |
| $q_{02}$ | Transition rate estimate from state 0 to 2 | 3.66 |
| $q_{03}$ | Transition rate estimate from state 0 to 3 | 3.29 |
| $q_{04}$ | Transition rate estimate from state 0 to 4 | 2.05 |
| $q_{10}$ | Transition rate estimate from state 1 to 0 | 0.12 |
| $q_{12}$ | Transition rate estimate from state 1 to 2 | 3.53 |
| $q_{13}$ | Transition rate estimate from state 1 to 3 | 1.58 |
| $q_{14}$ | Transition rate estimate from state 1 to 4 | 1.13 |
| $q_{20}$ | Transition rate estimate from state 2 to 0 | 2.88 |
| $q_{21}$ | Transition rate estimate from state 2 to 1 | 0.84 |
| $q_{23}$ | Transition rate estimate from state 2 to 3 | 4.81 |
| $q_{24}$ | Transition rate estimate from state 2 to 4 | 1.93 |
| $q_{30}$ | Transition rate estimate from state 3 to 0 | 0.68 |
| $q_{31}$ | Transition rate estimate from state 3 to 1 | 2.54 |
| $q_{32}$ | Transition rate estimate from state 3 to 2 | 0.59 |
| $q_{34}$ | Transition rate estimate from state 3 to 4 | 3.72 |
| $q_{40}$ | Transition rate estimate from state 4 to 0 | 1.46 |
| $q_{41}$ | Transition rate estimate from state 4 to 1 | 4.36 |
| $q_{42}$ | Transition rate estimate from state 4 to 2 | 2.58 |
| $q_{43}$ | Transition rate estimate from state 4 to 3 | 4.11 |

These input parameter values produced the lowest AIC values for the two, three, four and five state models.
