# Supplementary material for: Characterizing infectious disease progression through discrete states using hidden Markov models
Source: PLoS One. 2020 Nov 20;15(11):e0242683. doi: 10.1371/journal.pone.0242683 (PMC7678993; doi:10.1371/journal.pone.0242683)
Supplement: S2 Table — Parameter estimates for the two and three hidden disease models were generated using the modified Baum-Welch Expectation Maximization algorithm. (DOCX) [file pone.0242683.s002.docx]

S2 Table. Four and five state model parameter estimates.

| *Parameter* | *Description* | *4 state model* | *5 state model* |
| --- | --- | --- | --- |
| Four state models | | |  |
| $\pi_{0}$ | Probability of starting in state 0 | 0.448 | 1.728e-51 |
| $\pi_{1}$ | Probability of starting in state 1 | 0.196 | 0.211 |
| $\pi_{2}$ | Probability of starting in state 2 | 0.195 | 0.173 |
| $\pi_{3}$ | Probability of starting in state 3 | 0.160 | 0.427 |
| $\pi_{4}$ | Probability of starting in state 4 | -- | 0.190 |
| $\alpha_{0}$ | Gamma distribution shape parameter for state 0 | 17.67 | 81742 |
| $\alpha_{1}$ | Gamma distribution shape parameter for state 1 | 21.72 | 229.0 |
| $\alpha_{2}$ | Gamma distribution shape parameter for state 2 | 4923 | 19.09 |
| $\alpha_{3}$ | Gamma distribution shape parameter for state 3 | 336.8 | 19.29 |
| $\alpha_{4}$ | Gamma distribution shape parameter for state 4 | -- | 5055 |
| $\theta_{0}$ | Gamma distribution scale parameter for state 0 | 0.139 | 7.526e-5 |
| $\theta_{1}$ | Gamma distribution scale parameter for state 1 | 0.261 | 0.033 |
| $\theta_{2}$ | Gamma distribution scale parameter for state 2 | 0.002 | 0.271 |
| $\theta_{3}$ | Gamma distribution scale parameter for state 3 | 0.023 | 0.124 |
| $\theta_{4}$ | Gamma distribution scale parameter for state 4 | -- | 0.002 |
| $\alpha_{0}\theta_{0}$ | Gamma distribution mean for state 0 | 2.448 | 6.152 |
| $\alpha_{1}\theta_{1}$ | Gamma distribution mean for state 1 | 5.674 | 7.581 |
| $\alpha_{2}\theta_{2}$ | Gamma distribution mean for state 2 | 8.643 | 5.182 |
| $\alpha_{3}\theta_{3}$ | Gamma distribution mean for state 3 | 7.642 | 2.400 |
| $\alpha_{4}\theta_{4}$ | Gamma distribution mean for state 4 | -- | 8.646 |
| $q_{01}$ | Transition rate from state 0 to 1 | 2.373 | 6.419 |
| $q_{02}$ | Transition rate from state 0 to 2 | 1.561 | 5.271 |
| $q_{03}$ | Transition rate from state 0 to 3 | 3.263 | 3.628 |
| $q_{04}$ | Transition rate from state 0 to 4 | -- | 2.566 |
| $q_{10}$ | Transition rate from state 1 to 0 | 1.537 | 0.086 |
| $q_{12}$ | Transition rate from state 1 to 2 | 1.351 | 3.798 |
| $q_{13}$ | Transition rate from state 1 to 3 | 1.626 | 1.298 |
| $q_{14}$ | Transition rate from state 1 to 4 | -- | 1.057 |
| $q_{20}$ | Transition rate from state 2 to 0 | 3.036 | 1.997 |
| $q_{21}$ | Transition rate from state 2 to 1 | 0.838 | 0.784 |
| $q_{23}$ | Transition rate from state 2 to 3 | 3.755 | 3.683 |
| $q_{24}$ | Transition rate from state 2 to 4 | -- | 1.679 |
| $q_{30}$ | Transition rate from state 3 to 0 | 3.628 | 0.619 |
| $q_{31}$ | Transition rate from state 3 to 1 | 3.348 | 3.087 |
| $q_{32}$ | Transition rate from state 3 to 2 | 1.283 | 0.777 |
| $q_{34}$ | Transition rate from state 3 to 4 | -- | 4.209 |
| $q_{40}$ | Transition rate from state 4 to 0 | -- | 1.165 |
| $q_{41}$ | Transition rate from state 4 to 1 | -- | 4.681 |
| $q_{42}$ | Transition rate from state 4 to 2 | -- | 2.974 |
| $q_{43}$ | Transition rate from state 4 to 3 | -- | 3.633 |

Parameter estimates for the two and three hidden disease models were generated using the modified Baum-Welch Expectation Maximization algorithm
