# Supplementary material for: Characterizing infectious disease progression through discrete states using hidden Markov models
Source: PLoS One. 2020 Nov 20;15(11):e0242683. doi: 10.1371/journal.pone.0242683 (PMC7678993; doi:10.1371/journal.pone.0242683)
Supplement: S1 File — This section contains a detailed description of the Newton-Raphson Estimation of gamma emission parameters. (DOCX) [file pone.0242683.s003.docx]

**File S1. Description of gamma parameter estimation**

Newton-Raphson Estimation of gamma emission parameters.

MAP CFU emissions $x,$were modeled with gamma distributions, with the following pdf:

$$f\left( x | \alpha,\theta\right)=\frac{x^{\alpha-1}e^{-x/\theta}}{{\Gamma(\alpha)\theta}^{\alpha}}$$

And log likelihood function:

$$\log\left( L \right)=\Pi_{i=1}^{N}f\left( x | \alpha,\theta\right)= -Nlog\Gamma\left( \alpha\right)-\alpha Nlog\theta+\left( \alpha-1 \right)\log\left( \sum x_{i} \right)-\sum\frac{x_{i}}{\theta}$$

The maximum likelihood estimator for the scale parameter $\hat{\theta}$ is $\frac{\bar{x}}{\alpha}$, however there is no closed form maximum likelihood estimate for the shape parameter $\alpha.$Therefore, an estimate was generated using Newton Raphson estimation. The methods of moments estimator was used as the initial estimate for $\alpha:$

$$\alpha_{0}=\frac{Nx^{2}}{\sum_{i=1}^{N} \left( x_{i}-\bar{x} \right)^{2}}$$

Following the initial estimation of $\alpha_{0},$Newton Raphson Estimation was used to iteratively calculate $\hat{\alpha_{k}}$for each state k:

$$\alpha_{new}=a_{old}-\frac{\log\left( \alpha_{old} \right)-\psi\left( \alpha_{old} \right)-\log\left( \bar{x} \right)+\bar{logx}}{\frac{1}{\alpha_{old}}-\psi^{'}\left( \alpha_{old} \right)}$$

where $\bar{x_{k}}=\sum_{i} \frac{p_{i,k}x_{i}}{\sum_{k} p_{i,k}}$, $\bar{logx}=\sum_{i} \frac{p_{i,k}logx_{i}}{\sum_{k} p_{i,k}}$ and $p_{i,k}=\frac{f_{k}\left( i \right)b_{k}(i)}{\sum_{k} f_{k}\left( i \right)b_{k}(i)}$.

Estimation was stopped when the $\alpha$ value changed by less than 0.0001 or if the number of Newton-Raphson iterations exceeded 10000. If the number of iterations exceeded this threshold, the run was terminated.
